# Clinical Trial Design—A Review—With Emphasis on Acute Intervertebral Disc Herniation

**DOI:** 10.3389/fvets.2020.00583

**Published:** 2020-09-02

**Authors:** Nick D. Jeffery, Natasha J. Olby, Sarah A. Moore, The Canine Spinal Cord Injury Consortium (CANSORT-SCI), Sarah A. Moore

**Affiliations:** Author Affiliations: DACVIM-Neurology, Associate Professor, Neurology and Neurosurgery, Department of Veterinary Clinical Sciences, The Ohio State University College of Veterinary Medicine, Columbus, OH, United States; DACVIM-Neurology, Professor of Neurology/Neurosurgery; Distinguished Chair of Gerontology, Department of Clinical Sciences, North Carolina State University College of Veterinary Medicine, Raleigh, NC, United States; DACVIM-Neurology Professor; Chair, and Head, Department of Small Animal Clinical Sciences, College of Veterinary Medicine and Biomedical Sciences, Texas A&M University, College Station, TX, United States; DAVCIM (Neurology), Assistant Professor of Neurology, Purdue University College of Veterinary Medicine, Department of Veterinary Clinical Sciences, West Lafayette, IN, United States; Professor Neurology & Neurosurgery; Professor in Small Animal Clinical Sciences, College of Veterinary Medicine, Texas A&M University, College Station, TX, United States; ACVIM - Neurology Professor and Service Head, Neurology and Neurosurgery, Department of Veterinary Clinical Sciences, College of Veterinary Medicine, The Ohio State University, Columbus, OH, United States; Department of Clinical Sciences, Colorado State University, Fort Collins, CO, United States; Department of Clinical Science and Services, Royal Veterinary College, Hatfield, United Kingdom; The Royal Veterinary College, University of London, Hatfield, United Kingdom & CVS Referrals, Bristol Veterinary Specialists at Highcroft, Bristol, United Kingdom; Institute of Veterinary Pathology, Faculty of Veterinary Medicine, Leipzig University, Leipzig, Germany; Division of Clinical Neurology, Department for Clinical Veterinary Medicine, Vetsuisse Faculty, University of Bern, Bern, Switzerland; Department Small Animal Medicine and Surgery, University of Veterinary Medicine Hannover, Hannover, Germany/Europe; Neurology and Neurosurgery, Department of Veterinary Medicine and Surgery, University of Missouri, 900 E. Campus Dr., MU Veterinary Health Center, University of Missouri, Columbia, MO, United States; Department of Small Animal Medicine and Surgery, University of Veterinary Medicine Hannover, Hannover, Germany/Europe; ^1^Department of Small Animal Clinical Sciences, Texas A&M University, College Station, TX, United States; ^2^Department of Clinical Sciences, College of Veterinary Medicine, North Carolina State University, Raleigh, NC, United States; ^3^Department of Veterinary Clinical Sciences, The Ohio State University, Columbus, OH, United States

**Keywords:** explanatory, pragmatic, durotomy, glyburide, spinal cord injury

## Abstract

There is a clear need for new methods of treatment of acute disc herniation in dogs, most obviously to address the permanent loss of function that can arise because of the associated spinal cord injury. Clinical trials form the optimal method to introduce new therapies into everyday clinical practice because they are a reliable source of unbiased evidence of effectiveness. Although many designs are available, parallel cohort trials are most widely applicable to acute disc herniation in dogs. In this review another key trial design decision—that between pragmatic and explanatory approaches—is highlighted and used as a theme to illustrate the close relationship between trial objective and design. Acute disc herniation, and acute spinal cord injury, is common in dogs and there is a multitude of candidate interventions that could be trialed. Most current obstacles to large-scale clinical trials in dogs can be overcome by collaboration and cooperation amongst interested veterinarians.

## Introduction

Introduction of new medical interventions into everyday practice requires assessment of safety, effectiveness and, preferably, comparison with currently available therapies. These assessments are undertaken in the form of clinical trials. The typical process for clinical trial development follows a series of phases. Phase 0 trials involve a small number of subjects and often represent the first exposure of a drug or intervention to the target species, with the goal of learning how a drug interacts with the target. Phase I trials are typically “dose finding” studies that aim to define the optimum dose or regimen for therapy as well as delineating safety and defining toxicities associated with a new treatment in a healthy and, sometimes, a diseased population. Phase II and III trials have the goal of further establishing safety in the target population, assessing effectiveness or efficacy, and then validating those findings in larger populations of patients with the disease of interest.

Phase II and III randomized controlled trials (RCTs) are the “gold standard” for assessment of impact and are rigorously planned clinical experiments designed to minimize all sources of bias (1). There are many trial designs within this category, but the most common is the parallel group trial in which a population of affected participants is randomly allocated to receive either the novel intervention or the standard-of-care therapy (or placebo). The outcome of interest is then measured at a specified time after the intervention and the numbers or proportion of responders, or mean response, compared between groups. Although all clinical trial phases are important for therapeutic development in the context of spinal cord injury for dogs with intervertebral disc herniation, the focus of this review will be on Phase II and Phase III studies, with some mention of the Phase I studies in terms of the preparatory data they provide.

## Explanatory Vs. Pragmatic

Clinical trials can also be categorized according to whether they aim to be *explanatory* or *pragmatic*, and this distinction is important because it provides critical underpinning to the way that trials are designed. This dichotomy in aim is often overlooked in veterinary medicine but will provide a theme throughout this review (2). There is a range of criteria by which to decide whether a trial is pragmatic or explanatory (3) and many trials do contain aspects of both aims. However, fundamentally an explanatory trial aims to determine whether an intervention will work under ideal circumstances (i.e., “can this intervention work?”), whereas a pragmatic trial aims to determine if there is a benefit when it is applied in everyday practice (4). This difference has a multitude of secondary implications, most notably the tendency to strictly limit participant entry in explanatory trials and to restrict analysis to only those participants that completed every component of intervention and outcome measurement. On the whole, *pragmatic* trials are likely to have greater impact in everyday clinical medicine, because they have broader application.

There is often tension between these two approaches for veterinarians carrying out trials of interventions for spinal cord injury in dogs, because pragmatic trials would tend to be more useful for veterinary clinicians, whereas explanatory trials tend to be more useful in answering questions that arise from the “basic science” of spinal cord injury. In this context, an explanatory trial might tightly limit lesion location, injury severity, time since onset of injury and have detailed and complex outcome measures. All of these aspects might be matched with the tightly-controlled circumstances in which the intervention was previously applied in experimental animals, thereby providing a “proof of principle” that the intervention can translate from laboratory to clinic. Such a trial is more likely to find favor with a “basic science” spinal cord injury researcher. Nevertheless, researchers interested in developing interventions that can be translated from laboratory to clinic would also need to be cognizant that an intervention that works in a limited patient population under ideal circumstances and with a complex outcome measure used to detect benefit might not necessarily have useful clinical impact in humans—or pet dogs—with spinal cord injury. In contrast, a pragmatic trial might include all cases of thoracolumbar spinal cord injury and might focus its outcome measures on owner judgement of each animal's level of function and perceived quality of life. This type of trial will more likely find favor with veterinarians treating such cases, and also with researchers who have a strong interest in translational research: if an intervention can still show effectiveness even when used in sub-optimal circumstances it is likely to be sufficiently robust to translate into human spinal cord injury. The downside to this type of trial is that it may be difficult to understand why a treatment fails in a pragmatic trial; the loose inclusion criteria and outcome assessments may obscure a real effect that is lost in the noise of other competing effects.

Both explanatory and pragmatic trials have value and, in practice, many trials will incorporate aspects of both study aims; nevertheless, it is critical to consider these two, sometimes conflicting, aims during the design process.

## Key Elements Of A Randomized Controlled Trial

The classic design of Phase II/III RCT may be explanatory or pragmatic and has many prerequisites, notably *random* allocation of individuals to experimental and control groups, *concealment* of allocation before enrolment, assessment of follow-up by *blinded observers, pre-specified* definition of outcome assessment methods and comparisons and, usually, enrolment of *large groups* of participants (so as to be able to apply effective randomization). Best practices in the design of RCT have been formalized and published in human medicine under the recommendations of the CONSORT statement (http://www.consort-statement.org/), and this set of guidelines can also readily be applied to veterinary clinical trials.

An important ethical consideration before undertaking an RCT, is whether there is clinical “equipoise.” A state of equipoise exists if there is a balance of expert opinion between the two interventions that are being assessed in terms of their effectiveness, or if there is a degree of uncertainty across the field with respect to the efficacy of a particular intervention (5). For instance, although there is evidence that fenestration alone provides similar functional outcomes as decompressive surgery for “deep pain positive” pelvic limb paralysis or paresis following acute thoracolumbar intervertebral disc herniation (6), the expert consensus is that there IS a difference in outcome between these interventions. Therefore, a trial comparing these two options would not currently be considered ethical because expert opinion does not consider them equal in value. Although decompressive surgery has not been proven as the standard of care through a RCT, it has become so by default through synthesis of other types of evidence and through expert opinion.

For a variety of reasons, many aspects of RCTs can be difficult to achieve in veterinary medicine and, in general, there are few reported large-scale RCTs in animals [although note (7)]. However, a small number of clinical trials have already been carried out in dogs with acute and chronic spinal cord injury, most of which take an explanatory approach to trial design. Because one of the ultimate goals of CANSORT-SCI is to provide data on dogs with spinal cord injury that can lead to new therapies effective at the population level, the emphasis in this overview will be on construction of large-scale *pragmatic* trials, a design so far used less commonly in veterinary medicine but most likely to change how spinal cord-injured dogs are handled in future.

When considering an RCT there are several key questions to answer:

◦ *Does the trial aim to be explanatory or pragmatic?*
◦ *What population will be examined?*
◦ *What intervention will be applied?*
◦ *What will the comparator be?*
◦ *What outcome measure will be used?*
◦ *What degree of improvement will be detected? (Including its clinical impact)*.

## Selection of Cases

Although there is much to be learned about how best to treat dogs with spinal cord injuries of all types, those that have incurred acute thoracolumbar disc herniation are most in need of new therapies. This is partly because it is the most common type of injury (8), and partly because there is a recognized poor prognosis for dogs in some sub-categories of this cohort (9, 10). The main impetus that drives the perceived need for a new therapy for spinal cord injury in dogs is the lower proportion of dogs that recover locomotion (and other functions) after presenting with loss of “deep pain sensation” following acute thoracolumbar intervertebral disc herniation. In this sub-group, the proportion that recover independent quadrupedal locomotion is usually estimated to be around 55%, in contrast to the estimated 90–95% recovery for dogs that present with “deep pain sensation” intact (6, 10, 11). Furthermore, most of these deep pain negative dogs do not recover appropriate autonomic function either. It is currently a major source of frustration for owners and veterinarians alike that we cannot offer anything better for these patients and so this review will focus on this specific sub-set.

### Refining Inclusion Criteria

As well as having a clear clinical need for new therapy, dogs that present as “deep pain negative” can almost immediately be identified as potential trial candidates. However, although these cases can be rapidly recognized, it is important to note that this group is not homogenous. Such “deep pain negative” cases have variable duration and rate of onset, delay before presentation, severity of compression and inter-animal variation in body weight or conformation and so they can be further sub-divided if necessary, and this choice might be guided by deciding whether the trial has pragmatic or explanatory aims. For instance, it could be considered that a trial to investigate a putative therapy should be restricted to dogs that present within a specific time window and are of a specific age (i.e., leaning toward a more explanatory design). The advantage of investigating treatment effects in a sub-group of the whole population is that if they are more homogenous then the signal-to-noise ratio of any treatment effect can be more readily discerned. The drawback is that the proportion of cases within each sub-group will of course be smaller than the total population, so causing more difficulties with case recruitment adequate to achieve the prerequisite sample size.

Questions can also be asked about whether to restrict entry to a trial to specific types or sizes of dog. An important corollary is that, strictly, trial results only guide treatment of similar types of patient in the future. For instance if a drug for diabetes (in people) was successful in trials in obese males over 50 years old, there might be doubt about whether the results might apply to underweight 15 year old female patients. This aspect of clinical trial interpretation is known as the *generalizability* (1) and must be used to inform design. For dogs with spinal cord injury after intervertebral disc herniation the majority of cases will be middle-aged chondrodystrophic dogs and so there might be merit in restricting trial entry to these cases. The results could then be used to apply to the most commonly affected patients in future. On the other hand, if a 4 year-old German shepherd were to present with an acute herniation in future then we might not necessarily expect the same results as were obtained in the trial.

The alternative to restricting trial entry is to set up a more *pragmatic* trial, in which all-comers can be included. A possible drawback to more eclectic enrolment, especially when considering spinal cord injury in dogs, is that the trial arms easily become unbalanced through inclusion of relatively unusual cases (because they may randomize to one or other arm only), unless large numbers of cases are included. Another aspect of specific case selection that might apply regarding dog size is the widespread perception that recovery is different between large and small dogs (12). Again, if the treatment groups are sufficiently large this does not cause a problem—large dogs can be assumed to randomize equally to the two arms of the trial.

Although there is reason to think that deep pain negative dogs constitute the group most in need of new therapy, it could also be questioned whether there might be a need to investigate whether the recovery rate for dogs that are deep pain positive might also be enhanced. This enhanced recovery might take the form of a greater proportion recovering to walk or that the recovery could be made more rapid or more complete. Because of the inherent need for more complex outcome measures for these patients, it is likely that designs for studies on these dogs will be explanatory rather than pragmatic.

## Intervention

Selection of test interventions in clinical trials is usually based on pre-clinical data, which have generally been derived in experimental animals. When applied to human clinical trials, the steps toward a Phase II/III RCT would usually include a Phase 0 or Phase I “first-in-human” trial to assess toxicity and, depending on the nature of the intervention, often also include pharmacokinetic and pharmacodynamic studies to determine optimal dosing regimens. Appropriate surgical or physical therapy interventions are often less formally assessed at the pre-clinical stage because the relevant procedures may not be feasible or appropriate in experimental animal subjects. Traditionally, new therapeutic interventions in veterinary medicine are often derived from human medicine, but in spinal cord injury there are no therapies available for treatment of humans that are not available for dogs. In both species, treatment consists of care to maintain blood pressure, spinal cord decompression, and vertebral stabilization if appropriate, physical therapy and allowing plenty of time for nervous system recovery and plasticity (10, 13, 14).

There is a huge number of interventions that could potentially be applied to dogs with spinal cord injury, many of which have been extensively tested in laboratory animals over many years [e.g., (15–17)]. The decision as to which to select for further evaluation through RCTs in clinical cases might be determined by many factors, most notably knowledge about toxicity, the feasibility of appropriate dosing and the feasibility of application within a time period in which the agent has been shown to be effective. For instance, although tetrodotoxin can reduce the loss of spinal cord tissue and function after injury (18) it has very serious potential toxicity and was ineffective when applied 4 h after injury (19). Unfortunately, only about 15% of canine cases of spinal cord injury are presented to a specialty care facility within 8 h, with a much small proportion likely presented within 4 h; therefore, most cases cannot be treated at an appropriate specialist center within such a short period after injury (20). Alternatively, if we were to consider that suitable cases for a clinical trial were dogs that had chronic spinal cord injury—i.e., that they had an acute spinal cord injury from which they made an incomplete recovery—then the time period for the intervention becomes much less critical and a different series of intervention options is available.

Of the multitude of available medication interventions that might be useful based on reported success in laboratory animal models, many could plausibly be converted into clinical therapies in dogs. It is reasonable to consider that a credible subject for a RCT in dogs would be one that has shown benefits in experiments in more than one laboratory and more than one model of injury. Prominent examples of medications that meet these criteria and could be used acutely as neuroprotective strategies for spinal cord injury include riluzole (21), glyburide (22), and minocycline (17), all of which have been the focus of (Phase I or Phase II) clinical trials in people (see ClinicalTrials.gov). Again, it must be asked whether any of these agents can be applied rapidly enough after the injury in pet dogs, for which the median time to presentation is 24 h, to be beneficial (20).

In terms of surgical interventions, there is accumulating evidence that durotomy/duroplasty may be of value in reducing the intraparenchymal spinal cord pressure (thereby improving blood flow) in humans (23), experimental animals (24) and, recently, clinical canine patients (25, 26). This intervention has the benefit of being applicable for many hours, or even days, following an acute spinal cord injury and so could readily be translated into clinical veterinary practice. Currently there is clinical equipoise regarding this intervention, with closely balanced evidence for and against.

None of the interventions mentioned above are complicated to apply and so could all be used within pragmatic and explanatory trial frameworks.

## Comparator

The comparison therapy for dogs entered into a RCT to test an intervention for acute spinal cord injury would be “standard care,” which would consist of cross-sectional imaging and decompressive surgery (9, 10). Placebo therapy would not be a credible (nor ethical, see below) option in view of current clinical thinking and in some jurisdictions (the UK) is not permitted. Even so, “standard care” is not well-defined, especially in terms of anesthesia protocols, fluid therapy before and during surgery and, especially, post-operative care and physical therapy.

Therefore, other peri-operative therapies might require recording, or might require controlling through defined inclusion/exclusion criteria, when investigating a new intervention, although, again, the need to limit would be determined by how much the balance lay toward an explanatory approach. It is possible that some routine interventions might impact the results or interact with the trial therapy. There is some limited evidence that physical therapy for spinal cord-injured humans can have an impact on outcome (27, 28). Some data suggest benefits in dogs too (29), although a previous RCT on this subject did not support this conclusion (30). Nevertheless, at present there is no single protocol defined for physical therapy after decompressive spinal surgery in dogs, or which sub-population (if any) is most likely to derive benefit, and so this might need attention during a clinical trial. In large pragmatic trials it would not be necessary to define specific physical therapy protocols because the effects of any specific regimen would be expected to randomize equally between treatment groups amongst the large patient numbers, but this might not apply in smaller trials. Similarly, use of other drugs before and after surgery might also require controlling, or recording for inclusion as an analytical covariable. For instance, glucocorticoids have been examined for their effects in numerous studies (31), albeit without strong evidence of effect. More recently, opiates, specifically kappa opioids, have been implicated in worsening outcomes in experimental animals with spinal cord injury (32).

## Outcome Assessment

In every clinical trial it is imperative to define an outcome measure that will be used to define whether the intervention has achieved its goal of improving patient outcome. It is also necessary that the outcome be defined BEFORE the trial so as to prevent selective reporting after the data are collected, implying that it requires careful consideration. Useful outcome measures vary a great deal—between those that directly measure a key outcome that is definitely important to a participant, such as death—to those that are termed “*surrogate outcomes*” and measure something related to a key outcome but is not that key outcome—for instance, the area of MRI abnormalities in the brain of patients that have multiple sclerosis [e.g., (33)]. A pragmatic approach will use easily measured outcomes, which might be loosely defined, whereas explanatory trials will aim to quantify outcomes more precisely, often using relatively complex schemes or equipment.

### Specific Definitions Are Required

For spinal cord injury trials in dogs the most obvious outcome measure would be the ability to walk, especially since that is usually the lost function that encourages owners to seek veterinary advice. There is much merit in using this as an outcome measure, but there are some details that require attention when designing a trial. For instance, what does “ability to walk” mean: how many consecutive steps defines a dog as being “able to walk?” Does this apply to every walking surface? The most difficult aspect when recording the ability to walk after severe spinal cord injury in dogs is their ability to (sometimes) develop the ability to “spinal walk”—which is usually defined as the ability to walk without any clinical evidence of neural communication between the head and the limbs (i.e., clinically, dogs with persistent loss of deep pain sensation) [(29, 34) and see Lewis et al., companion article in this issue]. In terms of analysis of the effectiveness of a putative therapy, explanatory trials would wish to exclude recovery that is mediated by “spinal walking” because it does not reflect true regulation of locomotion from the brain and cervical spinal cord (i.e., the trial question is: “can the intervention restore the brain's ability to control the pelvic limbs?”). However, the pragmatic approach would be that if the dog ***can*** walk it does not matter whether the dog is able to voluntarily regulate this motion or not (i.e., the trial question is: “can the dog get around the yard?”). Both are acceptable outcomes but it is essential that these possibilities are considered before the trial and that the appropriate measure is used to address the question that is posed.

Recently the ability to “walk 10 (consecutive) steps” has increasingly been taken as a relatively pragmatic indication of recovery of locomotion [e.g., (35)]. Originally, this measure was used because many dogs that spinal walk are not able to take as many as 10 consecutive steps, although this does not encompass ALL spinal walking dogs (see Lewis et al., this issue) and there is also a question as to whether the flooring surface should be considered too. One advantage of using the 10 step “convention” is that by becoming a recognized standard the results of trials carried out on different interventions can be (broadly) compared. There is of course nothing special about 10 steps as opposed to 20 steps etc., but usually animals that can recover to walk 10 steps can then also go on to improve and walk further.

### Outcome Observer

The other question that has to be asked about a simple outcome measure such as walking, is who will make the final decision on the outcome? Will it be the owner or will it be a veterinarian, or a specialist veterinarian? The answers that are given by each may well be different. A specialist neurologist is much more likely to identify spinal walking compared to an owner, and they are much more likely to feel that the distinction is important. Nowadays it might also be asked about whether the outcome can be determined remotely: can the specialist running the trial make a determination of whether a dog can walk (or urinate voluntarily) by observing a video recording? Fortunately, recent work has suggested that it is relatively straightforward to train observers to a common outcome (36). A related question is the *blinding* of outcome observers. It is critical that those running the trial—and ideally owners too—are unaware of which treatment arm their dog has been allocated to. The people running the trial can reasonably be expected to be biased and owners might also interpret outcome in light of wishing to find that the new therapy is beneficial. Therefore, it is imperative that recording of outcome is done by someone who is unaware of the treatment allocation. This requirement can be problematic in veterinary medicine because of the limited personnel available in many clinics, including those trained to ask penetrating questions about voluntary function observed in the dogs, or to carry out more complex evaluations of function.

Although the ability to walk is an obvious and relevant outcome measure there are many other approaches to outcome assessment after spinal cord injury in dogs. In spinal cord injury research on experimental animals, locomotor scoring schemes have been applied for decades, most recently in the open-field “BBB scale” that grades the use of each pelvic limb and the coordination between thoracic and pelvic limb girdles (37). Similar schemes have been devised for use in dogs (38, 39) and all carry the advantage of allowing the ***quality*** of locomotion to be assessed, so implying that grades of recovery can be measured. However, there are also drawbacks, most notably that these scores are not truly numerical (and so are ordinal rather than continuous scales), which complicates interpretation (40), and there is also a great deal of inter-animal variability in outcome, even in rats that have incurred highly-regulated identical injuries (41). In addition, although easily applied in practice, these scales are designed to detect a surrogate outcome—one that is collected for the purposes of a trial rather than to detect a useful clinical benefit. The relationship between (small) improvement on these scales and clinical function is uncertain.

Kinetics and kinematics provide even more finely graded outcomes and kinematic analysis can be especially valuable because it can imply conduction across a lesion in the thoracolumbar area through detection of coordination of phase patterns of thoracic and pelvic limb stepping (42). These outcomes have been used to assess outcome in canine spinal cord injury trials because they are able to detect subtle changes in function (43, 44) that might realistically be expected to occur following an intervention in severely and chronically affected individuals. On the other hand, kinematic measures are clearly *surrogate* outcomes, especially when applied to animals walking on a treadmill, and it can also be argued that the detection of small differences in function might not have much clinical relevance. Again the choice here is outlined as a distinction between pragmatic (“can the dog walk?”) and explanatory (“is there a change in kinematics?”) outcomes and reiterate the need to select the outcome that is most appropriate for each individual trial.

Alternative outcomes, most notably bladder control, may also be usefully examined. Many owners nowadays are not all that concerned if their dog cannot walk, since they can be adequately mobile in carts, but they may be much more concerned about urinary control. There are many methods to define urinary control, ranging from the pragmatic (e.g., “does the dog urinate in the house?”) to more precise, but clinically remote, outcomes such as bladder compliance (the ability of the bladder to accommodate increasing urine volume). The use of bladder compliance as an outcome for a canine spinal cord injury trial has previously been described (44), although there are currently gaps in knowledge regarding normal bladder function in dogs (see companion article in this issue).

Finally, electrodiagnostic tests, particularly sensory and motor evoked potentials that measure long tract function, can be used as outcome measures (44). These are clearly *explanatory* outcomes and primarily used as an aid to interpreting mechanisms of change in function associated with an intervention.

## How Many Cases Will be Needed?

The number of participants needed to be enrolled in a clinical trial is determined by sample size calculations, which are determined by the desired power of the study and the false positive rate that is acceptable. There is also a difference in numbers needed depending on whether the sample size will be calculated based on a change in proportion reaching a specific criterion, or whether a specified difference in mean values is used. On the whole, power of clinical trials is set at 0.8 or 0.85 (*i.e*. β = 0.2 or 0.15) and the false positive value is usually set at 0.05 (*i.e*. α = 0.05). The methods for calculating sample size are widely available online and contained in various software packages as well as in numerous publications [MedCalc.net (45)].

Sample size calculations also depend on the size of the difference in outcome between groups that is being sought and the variability in the measure between and within individuals. Smaller differences in outcome and greater variability demand larger sample sizes. Variability in outcome can occur because of variability within and between individuals and can also result from imprecision in measurement.

If determining sample size based on proportions reaching a specified criterion (e.g., ability to walk 10 steps) it is necessary to have a reasonable estimate of the outcome after standard therapy (usually derived from previous publications) and to then estimate the proportion that might recover following the test intervention. This estimation is best derived from preliminary data, but can also be based on what might realistically be useful in the clinic, which might, in turn, depend on the invasiveness or toxicity of that intervention. On the whole, if the proportion of the sample that reach the criterion is close to 1 or 0 then sample sizes are much smaller than they are for proportions close to 0.5. For instance if we are to look at the proportion of deep pain dogs recovering to walk after standard therapy (~0.55) and consider that improving this to 0.65 would be clinically worthwhile, then the necessary sample size is ~375 per group (assuming two-sided testing with α = 0.05, β = 0.2). On the other hand, if we were interested in reducing the proportion of deep pain negative dogs that develop myelomalacia (current therapy is associated with ~0.15) to 0.05, then the sample size needed would be ~138 per group. This statistical efficiency provides a reason to be attracted to trials on very severely, and possibly chronically, injured individuals that are unlikely to recover spontaneously [see (46)].

Using the change in mean values between comparator groups is more statistically efficient, but requires a numeric outcome measure. It is also necessary to know (or estimate reasonably) the mean and standard deviation of the intervention and control groups. A problem that frequently arises is that it is difficult to know how much change in the outcome measure is meaningful. For instance, if we were to examine stride length as an outcome (as is commonly used in experimental rodents), how much change would be clinically meaningful for a paralyzed dog? The other aspect is that this method is highly dependent on the precision of measurement of the outcome. If there is a great deal of variability the ability to be sure that there is a real difference between groups is blunted. Similarly, if the outcome measure turns out not to be normally distributed many of the assumptions in analysis will be breached (although data transformation can often overcome this problem).

Whatever method is used the smaller the difference that is sought, the larger the sample size needed. When calculating sample size it is important that the sample size be realistic. Canine spinal cord injury is very common and so many hundreds of dogs can be accumulated, although that may require multicenter collaboration to achieve. On the other hand, it is also important that the difference between control and intervention group that is sought should be realistic. Most treatments in medicine have a moderate effect size and so, for instance, it is not realistic to power a study to detect a 50% difference in recovery between groups of deep pain negative dogs—no treatment is realistically going to be that effective.

Pragmatic vs. explanatory approaches can differ in terms of numbers needed in two main ways. First, an explanatory trial might be expected to refine the entry criteria with the aim of being able to discern even small differences between intervention and control and the sample size might then be reduced (although the selected cases will be a sub-set of the whole pragmatic population). Second, pragmatic trials tend to rely on “intention-to-treat” analysis in which all cases that enter the trial are included, irrespective of whether they received their allocated treatment or not. On the other hand, explanatory trials tend to rely on “per protocol” analysis, in which only participants that completely complied with the trial protocol are analyzed. Reliance on per protocol analysis tends to increase the numbers needed to achieve appropriate power because there will be many participants lost between enrolment and analysis.

## Analysis

Well-designed simple parallel group clinical trials usually do not require complicated statistical testing. Calculation of relative risk for a specific outcome [e.g., see CRASH trial (47)], chi-squared test or *t*-test (or non-parametric equivalent) is often sufficient to answer the basic trial question. Sometimes baseline measurement should be included as a covariate, necessitating use of analysis of covariance methods (usually implying regression techniques). Great care has to be taken with any sub-group analysis, and sub-groups should not be analyzed unless they are *pre-specified* and had relevant power calculations applied before trial commencement. Much harm has occurred in humans through misinterpretation of sub-group analysis (48). Similar considerations apply to analysis of whole study groups for which pre-trial sample size calculations are not available and for which study power is unknown (49).

Exceptions that might require more complicated analysis include the more complicated study designs such as crossover or factorial trials. However, crossover trials will rarely be appropriate for analyzing effects of interventions for spinal cord injury (especially acute injury, because time will be assumed to have a strong effect) and factorial trials require identification of interventions that might interact with each other (otherwise they have no advantage over parallel group trials) and there are few such combinations that have been identified in laboratory science.

## Ethical Considerations

The ethics of clinical trials in humans are complicated and rigorously regulated by international treaties and numerous guidelines (Helsinki Declaration: https://www.wma.net/policies-post/wma-declaration-of-helsinki-ethical-principles-for-medical-research-involving-human-subjects/). Those for veterinary clinical trials are less tightly regulated but follow the same basic principles: a trial needs to have a favorable risk/benefit analysis such that animals are carefully protected from detrimental consequences and have a reasonable chance of benefit from an intervention. Both of these aspects are usually addressed through investigations that precede the Phase II trial and may include data collection from experimental animals and Phase I studies in dogs. The question that inevitably follows is how much information is required before a Phase II/III trial can be considered reasonable and ethical. Similar questions have been asked regarding trials in human spinal cord injury (50) and the answer turns out to be different depending upon who is asked (51). On the whole, trials participants are more eager to press ahead with trials and researchers tend to be more cautious.

Beyond the requirement for preliminary data suggesting safety and efficacy, design of the trial itself most be undertaken ethically so as to ensure societal value of the information obtained. It is essential that all outcome measures can be assessed appropriately and that results of the study will be useful, which implies that the results will be disseminated to the scientific community by publication. Most clinical trial work in pet dogs requires ethical oversight from the Institutional Animal Care and Use Committee (IACUC) or a hospital review board at the center that is coordinating the trial, plus informed caregiver consent. Study review and approval generally includes assessment of design and proposed outcomes, component analysis of protocol activities that extend beyond the standard of care and how risks associated with those are mitigated, examination of sample size calculations, and investigator and personnel credentials and training in animal care.

## Practical Obstacles to Clinical Trials in Spinal Cord Injury in Dogs

There is a strong rationale for clinical trials on spinal cord injury treatments in dogs and many have been carried out and are readly accessible via PubMed (or other) searches using the keywords “dog” “spinal cord injury” “trial.” It is readily apparent that few fulfill all the appropriate requirements for an effective clinical trial and many have design problems, such as lack of a control group (or unclear controls), that limit their value. This current review article is not intended to be a systematic review of previously published trials for but instead provides a checklist of items that can be applied by readers to evaluate each of the previously published trials themselves. On the whole, the most major problem has been the inability to recruit a sufficiently large number of participants, mainly because trials have been located at single centers. Challenges in multicenter trial design include: logistical challenges of coordinating fee structures, study review and approval across different sites; additional costs incurred by incorporation of subawards into funding applications; lack of availability and consistent infrastructure and equipment across sites; need for reasonably consistent training and application of trial methods at each center; barriers assumed to exist with incorporation of trial methodologies into routine private referral practice and the assumed traditional independent-mindedness of veterinarians in general. Retention of recruited clients can also be problematical, especially for spinal cord injury, for which prolonged follow-up is essential. Many previously published clinical trials in canine spinal cord injury are discussed in more detail in the companion article in this issue “*Ambulation in dogs with absent pain perception after acute thoracolumbar spinal cord injury*.”

The main question is, how can obstacles to multicenter trials be overcome? Answers might include simplifying trial procedures so that many individuals and centers can be involved, establishing groups with an appropriate democratic structure to allow all collaborators to feel valued and involved in decision-making and, importantly, securing funding that bonds groups together and enables employment of trial-specific personnel.

## Possible Clinical Trial Designs in Acute Spinal Cord Injury in Dogs: Comparing and Contrasting Pragmatic and Explanatory Examples (Table 1)

The focus of this review article is on clinical trials for treatment of dogs with thoracolumbar spinal cord injury resulting from acute disc herniation. Whilst there are strong arguments for carrying out trials in dogs that have reached an unacceptable plateau of recovery at a later date—because they definitely require new therapies and the effect of new therapies might be more easily detected (because the baseline recovery rate is so poor)—here we will focus on two possible candidates for trials for dogs that present with loss of “deep pain sensation” in the acute phase. We have selected two candidate interventions that are “ready to go” but have constructed contrasting trial designs to highlight the many choices that need to be made. Although we describe here an explanatory trial focused on glyburide and a pragmatic trial focused on durotomy these specific approaches do not need to be specifically linked in this way to these interventions.

**Table 1:** Outline of two contrasting example approaches to spinal cord injury trials in dogs.

	**Explanatory**	**Pragmatic**
Population	Chondrodystrophic dogs weighing <15 kg with 7 vertebral-length gap on myelo MRI and serum GFAP >7 ng/mL, presenting within 24 h of when last seen walking	Any deep pain negative dog
Intervention	Glyburide 150 μg/kg, then 75 μg/kg TID for 3 days	4-vertebral length durotomy
Comparator	Sham tablets prepared by pharmacy	Standard care
Outcome	Open-field score repeated at weekly intervals for 3 months	Death or euthanasia by 3 weeks
Other restrictions	Physical therapy applied to animals in both groups for 30 min each day up till 3 months	No rules on physical therapy and adjunctive treatment
Personnel requirements	Training in open-field score analysis. Monitoring of dogs 24/7 for signs of hypoglycemia	No specific requirements
Analysis	Regression/ANOVA to analyze time x treatment interaction effects on open-field score over 3 month period	Chi-squared test to compare proportion of all-cause deaths between groups
Animals included in analysis	Only those that received glyburide at appropriate dose and times for 3 days, received physical therapy according to protocol and have follow-up for all time points until 3 months	All cases; analyzed according to intention-to-treat
Sample size calculation (approximate)α = 0.05β = 0.8	Expecting a score of 10.7 in control group and 12 in the treated group, with SD of 1.5, would require 22 per group	Expecting 15% deaths at 3 weeks in controls and 5% in intervention group would require 138 per group
Likelihood of a positive outcome changing clinical practice	Minimal	Very high

*(a) Glyburide*, aka glibenclamide, is a hypoglycemic agent that was used as an aid to controlling diabetes mellitus in people. It also has effects on the Sur1/TrpM4 channel that is involved in the progression of spinal cord tissue damage after acute contusion and its beneficial effects after spinal cord injury have been well-documented in several neuroscience laboratories throughout the world (52). Recently, pharmacokinetic studies have been carried out in dogs, showing that it has a good safety margin (hypoglycemia was not a problem at the doses needed to attain appropriate serum concentrations) and allowing construction of an appropriate dosing regimen for treatment of spinal cord injury (53). This drug therefore appears to have many advantages: it is widely available as a standard commercial preparation, it is cheap, the pharmacokinetics and safety are acceptable for use in dogs and it has shown benefit in many pre-clinical studies of spinal cord injury.

However, one question regarding glyburide is whether we would need to have a time limit on when trial dogs become paraplegic before presentation. In experimental work with glyburide, it would appear that it is most beneficial if it can be given before about 8 h after injury (54). It can be problematical to know for how long dogs have been paraplegic when the owners find them and it can be problematical to get a dog into a specialist clinic for treatment with a trial drug within the 8-h period. Also, if we were to limit inclusion to dogs that presented within 8 h of injury it would greatly reduce our expected recruitment numbers. So the best plan may be to design the trial to accept any dog that has become paraplegic and deep pain negative within 24 h, with a pre-specified analysis of the sub-groups that present <12 and 12–24 h after paralysis.

*(b) Durotomy* has been posited as a treatment for acute spinal cord injury since it was first modeled in animals in the early twentieth century (55). Since then there have been several studies suggesting that it is, or is not, helpful in dogs with acute spinal cord injury (56–58). The great advantage that durotomy appears to have is that, according to experimental data, the effect of incising the dura persists over at least 3 days after the injury (24), therefore perhaps making it more appropriate than glyburide for translation into dogs. There is also the advantage that anyone who is doing the decompressive surgery can carry out this procedure without having to have additional study materials or equipment.

Although there has been debate over the value of durotomy as an additional decompressive technique that might aid in restoring blood supply (and therefore retaining tissue integrity) after spinal cord injury, there are now data supporting its efficacy in clinically paralyzed dogs (25, 26).

### How Might Dogs Be Randomized?

In large human clinical trials it is routine to use a central telephone service that designates treatment to each patient as they are enrolled. This method facilitates multicenter participation but does demand high level staffing and funding. For many veterinary trials for which funding at high levels (if at all) is unavailable, simple randomization that is blocked by center (i.e., each center has their own randomization) is still possible. The most straightforward way to randomize is to prepare a set of opaque envelopes, each containing the treatment allocation on a slip of paper and made up in variable-sized batches (so that the allocating clinician cannot predict what treatment the next patient will receive when they get near the end of a batch). The same procedure can be duplicated at each participating center; it is important that each center should randomly assign cases independently, to ensure that one center does not allocate unevenly compared to another. It is essential that clinicians who will allocate animals to treatment cannot know which intervention each patient will receive until they open the envelope (i.e., there is appropriate *allocation concealment*). Numbering of envelopes also prevents allocations being selected.

### How Might We Measure Outcome?

In these contrasting trials we have selected contrasting outcomes for the two interventions. For the explanatory trial on glyburide we have suggested using an open-field outcome assessment of walking (38) that is applied at weekly intervals for 3 months. These scores can then be assessed using repeated measures ANOVA or equivalent regression analysis, preferably accounting for the non-numerical nature of the outcome scores; animals that die or are euthanased before 3 months will be excluded from this analysis. For the durotomy trial we are interested in possible effect of averting progressive myelomalacia and so the simplest outcome to measure is whether dogs survive for longer then 3 weeks (although this will inevitably include some dogs euthanased for reasons apart from myelomalacia). The proportion surving for longer than 3 weeks will be compared between treatment groups using Fisher's exact test.

### Ethical Considerations

A question might also be asked about whether it can be regarded as ethical to carry out a trial if preliminary studies show support for efficacy of an intervention—most notably here regarding durotomy. On the other hand, there may also be long (or even short-term) adverse effects of the new intervention that have not yet been detected and, furthermore, a principle of evidence-based medicine is that new therapies should be rigorously tested before widespread clinical adoption. It is also essential that all participating centers and personnel are adequately trained and equipped to carry out the trial procedures; training videos can often be used to facilitate such preparation.

Another way of looking at the assumed outcomes of a successful trial on durotomy is that if there really is an improvement in outcome from 15 to 5% that are dead by 3 weeks then this would suggest a number-needed-to-treat of 100/10 = 10, i.e., for every 10 animals that are treated by durotomy only one additional animal survives for more than 3 weeks. While this can be justified if the therapy is beneficial it does also mean that there is little reason to be too concerned about allocating an animal to standard care alone, especially considering that there may also be adverse effects of the novel intervention (see above).

Another aspect is that an independent data and safety monitoring committee should be established to oversee data and, sometimes, to carry out interim analysis as it accumulates. In the trials outlined in Table 1 this would only be realistic for the pragmatic trial on durotomy (because the other trial will enroll such a small number of cases). Such committees often use statistical stopping rules to aid decisions on interim analyses, to prevent a trial from continuing for too long if there is an unexpected but obvious imbalance in outcomes—an excess of benefit or of harm—before full trial recruitment has occurred (59). However, stopping rules can be controversial because stopping too early can lead to erroneous conclusions—especially with a bias toward larger effect size—or fuel continuing dispute regarding efficacy. One solution is to accept only extreme differences between groups at early stages, with progressive relaxation during the trial (60); the risks of multiplicity must also be incorporated. A more nuanced, and modern, option is to interpret interim results as a whole, taking into account statistics on both primary and secondary outcomes, the relative risk benefit and the problems that might arise in association with stopping too early, in order to provide evidence that is “beyond reasonable doubt” (61).

## Conclusions

Large *pragmatic* clinical trials to determine the optimal methods for treating dogs with spinal cord injury are undoubtedly required. The relatively poor outcome associated with severe (i.e., “deep pain negative”) thoracolumbar spinal cord injury following acute intervertebral disc herniation is the most obvious target. On the one hand, trials on such cases should mean that any “signal” resulting from an intervention will be easy to detect (because so little is expected of them); on the other, these cases are the hardest nuts to crack and so it is less probable that a detectable effect will be observed. Trials in less severely affected animals produces the opposite problem: many cases will get better anyway and so the signal of the intervention is lost in the noise of spontaneous recovery. Similar considerations apply to use of alternative outcome measures, including those used to examine autonomic function. There are many candidate therapies that could reasonably be tested and, worldwide, there are many affected dogs available for recruitment. Current barriers are largely problems of our (i.e., veterinarians') own making and can feasibly be overcome.

## Author Contributions

Subject of review was discussed between NJ, NO, and SM. NJ wrote first draft. All authors contributed to the input and approved the final version.

## Conflict of Interest

The authors declare that the research was conducted in the absence of any commercial or financial relationships that could be construed as a potential conflict of interest.
